# Exact solutions of $\kappa$-dependent Schrödinger equation with quantum pseudo-harmonic oscillator and its applications for the thermodynamic properties in normal and superstatistics

**DOI:** 10.1038/s41598-023-28973-7

**Published:** 2023-02-06

**Authors:** Uduakobong S. Okorie, Akpan N. Ikot, Ituen B. Okon, Lewis F. Obagboye, Ridha Horchani, Hewa Y. Abdullah, Karwan W. Qadir, Abdel-Haleem Abdel-Aty

**Affiliations:** 1 Department of Physics, Akwa Ibom State University, Ikot Akpaden, P.M.B. 1167, Uyo, Nigeria; 2 Theoretical Physics Group, Department of Physics, University of Port Harcourt, Choba, Nigeria; 3 Theoretical Physics Group, Department of Physics, University of Uyo, Uyo, Nigeria; 4 Theoretical Physics Programme, National Mathematical Centre, Abuja, Nigeria; 5 Department of Physics, College of Science, Sultan Qaboos University, P. C., 123, Al-Khod, P.O. Box 36, Muscat, Sultanate of Oman; 6 Physics Education Department, Faculty of Education, Tishk International University, 44001 Erbil, Iraq; 7 Department of Physics, College of Education, Salahaddin University-Erbil, Erbil, 44002 Kurdistan Region Iraq; 8 Department of Physics, College of Sciences, University of Bisha, P. O. Box 344, Bisha, 61922 Saudi Arabia

**Keywords:** Chemistry, Physics

## Abstract

The effects of the curvature parameters on the energy eigenvalues and thermodynamic properties of quantum pseudoharmonic oscillator are investigated within the framework of nonrelativistic quantum mechanics. By employing Nikiforov-Uvarov method, the energy spectra are obtained and used to study the ordinary statistics and *q*-deformed superstatistics as a function of temperature in the presence and absence of the curvature parameters. It is shown that the *q*-deformed supertatistics properties of the quantum pseudoharmonic oscillator reduce to the ordinary statistical properties in the absence of the deformation parameter. Finally, our results are illustrated graphically to show the behaviour of the energy spectra and thermodynamic properties for the three curvature parameters:$\kappa = - 1,\,\,\kappa = 1\,\,{\text{and}}\,\,\kappa = 0$.

## Introduction

In 1940, Schrödinger initiated the study of quantum systems on curved spaces^1^, using the factorization formalism. This study sparked further investigations and many authors started investigating the quantum system on curved spaces both in negative and positive curvatures in spherical geometry^2–4^. Later on, different studies were carried out on hyperbolic space, specifically on quantum harmonic oscillator by employing the geodesic spherical coordinates^5–11^. In all these considerations, the curvature parameter, $\kappa$ and its influence on the system considered was the focal point. Harmonic oscillator has been employed over the decades to study atomic vibrations in different molecular systems^12^ both in the relativistic and nonrelativistic regimes. Details of the studies on harmonic oscillator can be found in Ref.^13^ and other literatures on the reference list. Different authors have engaged various methods to evaluate the thermodynamic functions of different potential functions before now^14–21^. In recent times, macroscopic theories of these thermodynamic functions are much explained using abstract microscopic statistical mechanics^22^.

The concept of thermodynamic studies was later generalized by Beck and Cohen^23,24^, using the phenomenon called superstatistics. In this formalism, two different statistics were superimposed to explain non-equilibrium systems^25–27^. Much study have been carried out with superstatistics within equilibrium and non-equilibrium statistical mechanics framework^28–32^. The effects of cosmic-string parameters on harmonic oscillator have been considered using both ordinary statistics and superstatistics formalism^33^. Most recently, Edet and Ikot^34^ studied some diatomic molecules with shifted Deng-Fan potential, by employing the *q-*deformed superstatistics approach. In addition, Hassanabadi et al.^35^ employed the superstatistics formalism to study the effects of harmonic oscillator potential parameters with Dunkl derivative on thermodynamic functions. Their results were all reduced to the ordinary statistical mechanics as limiting cases. Three different types of superstatistics were comparatively studied by Chung et al.^36^. Here, internal energies for continuous and quantum discrete energies were considered as regards magnetic and paramagnetic models. As mentioned earlier, much research work has been carried out on superstatistics and thermodynamic properties, however, we decided to study both the superstatistics and normal statistics of pseudoharmonic oscillator using $\kappa$-dependent Schrödinger wave equation which is different from the conventional Schrödinger equation.

In this study, we shall first obtain the $\kappa$-dependent energy eigenvalues expression for quantum pseudo-harmonic oscillator within the framework of non-relativistic quantum mechanics. Thereafter, the energy eigenvalues expression obtained will be used to deduce the $\kappa$-dependent thermodynamic function expressions for the quantum pseudo-harmonic oscillator. In addition, the $\kappa$-dependent superstatistics properties of the quantum pseudo-harmonic oscillator will be obtained as well. We can confirmed to the best of our knowledge that no studies have been undertaken or reported concerning the influence of the curvature parameter on ordinary statistics and superstatistics of quantum pseudo-harmonic oscillator.

The article is organized as follow: In Section “[Sec Sec2]”, the $\kappa$-dependent Schrödinger equation with the quantum pseudoharmonic oscillator is solved and its energy spectra expressions in the presence and absence of the curvature parameters obtained. In Section “Evaluation of normal thermodynamic properties”, the closed form thermodynamic properties expressions of quantum pseudoharmonic oscillator are deduced for both $\kappa \,\, \ne \,\,0\,\,\,{\text{and}}\,\,\,\kappa \,\, = \,\,0$. The superstatistics properties of the quantum pseudoharmonic oscillator are evaluated in Section “Evaluation of thermodynamic properties of superstatistics”, using the modified Dirac delta distribution formalism, both in the presence and absence of the curvature parameters. The discussion of the results obtained is presented in Section “Results and discussion”. Section “Concluding remarks” finally gives the concluding remarks.

## $\kappa$-dependent Schrödinger equation solutions of quantum pseudoharmonic oscillator

The $\kappa$-dependent radial Schrödinger equation is defined as^11^

1 $$(1\, - \,\kappa \,r^{2} )\frac{{d^{2} R(r)}}{{dr^{2} }}\,\, + \,\,\frac{{(2\, - \,3\kappa \,r^{2} )}}{r}\frac{dR(r)}{{dr}}\,\, + \,\,\frac{2\mu }{{\hbar^{2} }}\left[ {E_{nl} \,\, - \,\,V\,(r)\,\, - \,\,\frac{{l(l + 1)\hbar^{2} }}{{2\mu r^{2} }}} \right]R\,(r)\,\, = \,\,0.$$

Here, $E_{nl}$ represents the energy eigenvalues of the $\kappa$-dependent quantum pseudo-harmonic oscillator, $\mu$ is the reduced mass and $V_{\kappa } \,(r)$ is the $\kappa$-dependent quantum pseudo-harmonic oscillator, which is defined as

2 $$V_{\kappa } \,(r)\,\, = \,\,\frac{{A\,r^{2} }}{{1\, - \,\kappa \,r^{2} }}\,\, + \,\,\frac{{B\,(1\, - \,\kappa \,r^{2} )}}{{r^{2} }}\,\, + \,\,C,$$

where $A,\,\,B\,\,{\text{and}}\,\,C$ are potential parameters. The curvature parameter $\kappa$ can either be greater than zero (spherical space), equal to zero (Euclidean Space) or less than zero (hyperbolic Space).

Substituting Eq. (2) into Eq. (1) gives

3 $$(1\, - \,\kappa \,r^{2} )\frac{{d^{2} R(r)}}{{dr^{2} }}\,\, + \,\,\frac{{(2\, - \,3\kappa \,r^{2} )}}{r}\frac{dR(r)}{{dr}}\,\, + \,\,\left[ \begin{gathered} \frac{{2\mu E_{nl} }}{{\hbar^{2} }}\,\, - \,\,\frac{2\mu A}{{\hbar^{2} }}\frac{{r^{2} }}{{(1\, - \,\kappa \,r^{2} )}}\,\, \hfill \\ - \,\frac{2\mu B}{{\hbar^{2} }}\frac{{(1\, - \,\kappa \,r^{2} )}}{{r^{2} }}\,\, - \,\,\frac{2\mu C}{{\hbar^{2} }}\,\, - \,\,\,\frac{l(l + 1)}{{r^{2} }} \hfill \\ \end{gathered} \right]R\,(r)\,\, = \,\,0.$$

With the help of the coordinate transformation $s\,\, = \,\,\kappa \,r^{2}$, Eq. (3) reduces to the following:

4 $$\frac{{d^{2} R(s)}}{{ds^{2} }}\,\, + \,\,\frac{{({\raise0.7ex\hbox{$3$} \!\mathord{\left/ {\vphantom {3 2}}\right.\kern-0pt} \!\lower0.7ex\hbox{$2$}}\, - \,2s)}}{s(1\, - \,s)}\frac{dR(s)}{{ds}}\,\, + \,\frac{1}{{s^{2} (1\, - \,s)^{2} }}\,\left[ \begin{gathered} (\varepsilon_{nl}^{2} \, - \,P_{1} \, - \,P_{2} \, - \,P_{3} )s^{2} \,\, \hfill \\ + \,\,(P_{4} \, - \,P_{3} \, + \,2P_{2} \, - \,\varepsilon_{nl}^{2} )s - \,(P_{2} \, + \,P_{4} ) \hfill \\ \end{gathered} \right]R\,(s)\,\, = \,\,0.$$

Here, the following abbreviations are defined:

5 $$- \,\varepsilon_{nl}^{2} \,\, = \,\,\frac{{\mu E_{nl} }}{{2\hbar^{2} \kappa }};\,\,P_{1} \,\, = \,\,\frac{\mu A}{{2\hbar^{2} \kappa^{2} }};\,\,P_{2} \,\, = \,\,\frac{\mu B}{{2\hbar^{2} }};\,\,P_{3} \,\, = \,\,\frac{\mu C}{{2\hbar^{2} \kappa }};\,\,P_{4} \,\, = \,\,\frac{l(l\, + \,1)}{4}.$$

We employ the Nikiforov-Uvarov (NU) method^37^ in Eq. (4), where the details are outlined in the ref.^37^ and the references therein. Hence, the analytical form of the energy eigenvalues for $\kappa$-dependent quantum pseudoharmonic oscillator is obtained as

6 $$\begin{gathered} E_{nl}^{\kappa \, \ne \,\,0} \,\, = \,\,\frac{{2\hbar^{2} \kappa }}{\mu }\left( {n\, + \,\frac{1}{2}} \right)^{2} \,\, + \,\,\frac{{2\hbar^{2} }}{\mu }\left( {n\, + \,\frac{1}{2}} \right)\left( {\sqrt {\kappa^{2} \, + \,\frac{2\mu A}{{\hbar^{2} }}} \,\, + \,\,\sqrt {\kappa^{2} \, + \,\frac{{2\mu B\kappa^{2} }}{{\hbar^{2} }}\,\, + \,\,l(l\, + \,1)\kappa^{2} } } \right)\,\, \hfill \\ \,\,\,\,\,\,\,\,\,\,\,\, + \,\,\frac{{2\hbar^{2} }}{\mu }\left( {\sqrt {\kappa^{2} \, + \,\frac{2\mu A}{{\hbar^{2} }}} \,\sqrt {\frac{{\kappa^{2} }}{4}\, + \,\frac{{\mu B\kappa^{2} }}{{2\hbar^{2} }}\,\, + \,\,\frac{l(l\, + \,1)}{4}} } \right)\,\, + \,\,\frac{{2\hbar^{2} }}{\mu }\left( {\frac{\kappa }{4}\, + \,\frac{\mu C}{{2\hbar^{2} }}\,\, + \,\,\frac{l(l\, + \,1)\kappa }{4}} \right) \hfill \\ \end{gathered}$$

When $\kappa \,\, = \,\,0$, Eq. (6) reduces to the energy spectrum for the standard pseudoharmonic oscillator as

7 $$E_{nl}^{\kappa \, = \,0} \,\, = \,\,C\,\, + \,\,\sqrt {\frac{{2\hbar^{2} A}}{\mu }} \left( {2n\, + \,1\, + \,\sqrt {1\, + \,\frac{2\mu B}{{\hbar^{2} }}\, + \,l(l\, + \,1)} } \right).$$

## Evaluation of normal thermodynamic properties

To determine the thermodynamic properties of the quantum pseudoharmonic oscillator, we first evaluate its partition function defined as^16–19^,

8 $$Z\left( \beta \right) = \sum\limits_{n\,\, = \,\,0}^{\infty } {e^{{ - \,\,\beta \,E_{n} }} } ,\,\,\beta = \left( {k_{B} T} \right)^{ - 1} ,$$

where $k_{B}$ is the Boltzmann constant and $E_{n}$ is energy of the nth bound state. Substituting Eqs. (6) and (7) into Eq. (8), we obtain the following respective expressions for partition functions:

9 $$Z^{\kappa \, \ne \,0} = \frac{1}{{2\sqrt {H\kappa \beta } }}\left( \begin{gathered} \left( {Exp\left[ { - \,\left( {HG_{1} G_{3} \, + \,2HG_{4}^{2} } \right)\beta } \right]\, + \,\frac{{H^{2} \left( {G_{1} \, + \,G_{2} } \right)^{2} \beta }}{2H\kappa }} \right) \times \hfill \\ \left( {\sqrt {\frac{\pi }{2}} \,Erf\left( {\frac{{\left( {\kappa \, + \,G_{1} \, + \,G_{2} } \right)H\beta }}{{\sqrt {2H\kappa \beta } }}} \right)} \right) \hfill \\ \end{gathered} \right).$$

10 $$Z^{\kappa \, = \,0} = \frac{{Exp\left[ { - \left( {C + G_{5} + G_{3} G_{5} } \right)\beta } \right]}}{{2G_{5} \beta }}.$$

Here,

11 $$\begin{gathered} G_{1} \, = \,\,\sqrt {\kappa^{2} \, + \,\frac{2\mu A}{{\hbar^{2} }}} \,;\,\,G_{2} \, = \,\,\sqrt {\kappa^{2} \, + \,\frac{{2\mu B\kappa^{2} }}{{\hbar^{2} }}\,\, + \,\,l(l\, + \,1)\kappa^{2} } \,;\,\,G_{3} \, = \,\,\kappa G_{2} ;\,\, \hfill \\ G_{4} \, = \,\,\left( {\frac{\kappa }{4}\, + \,\frac{\mu C}{{2\hbar^{2} }}\,\, + \,\,\frac{l(l\, + \,1)\kappa }{4}} \right)\,;\,\,G_{5} \, = \,\,\sqrt {2HA} \,;\,\,H\, = \,\,\frac{{\hbar^{2} }}{\mu } \hfill \\ \end{gathered}$$

Other thermodynamic properties are obtained using the following expressions:

12 $$\left. \begin{gathered} F\left( \beta \right) = - \frac{1}{\beta }\ln Z\left( \beta \right);\,\,U\left( \beta \right) = - \frac{d\ln Z\left( \beta \right)}{{d\beta }}, \hfill \\ S\left( \beta \right) = \ln Z\left( \beta \right)\,\, - \,\beta \frac{d\ln Z\left( \beta \right)}{{d\beta }};\,\,C_{v} \left( \beta \right) = \beta^{2} \frac{{d^{2} \ln Z\left( \beta \right)}}{{d\beta^{2} }}\,\, \hfill \\ \end{gathered} \right].$$

## Evaluation of thermodynamic properties of superstatistics

Superstatistics is known to be a superposition of different statistical models in statistical physics, which helps in studying non-linear and non-equilibrium systems^23,24^. By taking a Laplace’s transform of the probability density function $f\left( {\beta^{\prime},\beta } \right)$ within the concept of superstatistics, one can obtain the generalized Boltzmann factor defined as^31,32^

13 $$B(E) = \int_{0}^{\infty } {e^{{ - \beta^{\prime}E}} } f\left( {\beta^{\prime},\beta } \right)d\beta^{\prime}.$$

The probability density function obeys the normalization condition $f\left( {\beta^{\prime},\beta } \right) = \delta \left( {\beta^{\prime} - \beta } \right)$. Thus, the effective Boltzmann factor for the modified delta distribution function is given as^31,32^,

14 $$B_{q} (E) = e^{ - \beta E} \left( {1 + \frac{q}{2}\beta^{2} E^{2} } \right).$$

Here, *q* is the deformation parameter which lies between $0 \le q \le 1$, $E$ is the energy level of the system. It can be seen in Eq. (14) that when $q \to 0$, the superstatistics reduced to ordinary statistic mechanics^31,32^. Within the superstatistics, the thermodynamics functions of the system are valid for all values of *q* and all the thermodynamic functions as well as the energy depend on the system.

Thus, the partition function within the superstatistics formalism is defined as^34^,

15 $$Z_{q} \,\left( \beta \right)\, = \sum\limits_{n = 0}^{\infty } {B_{q} \,\left( E \right)} .$$

Hence, the partition function for the modified delta distribution can be written as^34^

16 $$Z_{q} \left( \beta \right)\,\, = \,\,\int\limits_{0}^{\infty } {B_{q} \,\left( E \right)\,dn} .$$

By substituting Eqs. (6) and (7) into Eq. (16) and employing the Mathematica software^38^, we obtain the q-deformed partition function in superstatistics for the quantum pseudo-harmonic potential, both in the absence and presence of the kappa parameter, respectively as

17 $$Z_{q}^{\kappa \, = \,0} \left( \beta \right)\, = \,\frac{1}{{4G_{5} \beta }}\left( {e^{{ - \,\left( {C\, + \,G_{5} \, + \,G_{3} G_{5} } \right)\beta }} \left( {2\, + \,q\left( {2\, + \left( {C\, + \,G_{5} \, + \,G_{3} G_{5} } \right)\beta \left( {2\, + \,\left( {C\, + \,G_{5} \, + \,G_{3} G_{5} } \right)\beta } \right)} \right)} \right)} \right).$$

18 $$\begin{gathered} Z_{q}^{\kappa \, \ne \,0} \left( \beta \right)\, = \,\frac{1}{{32\kappa^{2} \sqrt {H\kappa \beta } }}e^{{ - \,\frac{1}{2}H\left( {\kappa \, + \,2\left( {G_{1} \, + \,\,G_{2} \, + \,G_{1} G_{3} \, + \,2G_{4}^{2} } \right)} \right)\beta }} \hfill \\ \left( \begin{gathered} 2\left( {\kappa \, + \,G_{1} \, + \,G_{2} } \right)q\sqrt {H\kappa \beta } \left( {3\kappa \, + H\left( {\kappa^{2} \, - \,\left( {G_{1} \, + \,G_{2} } \right)^{2} \, + 2\kappa \left( {G_{1} \, + \,G_{2} \, + \,2G_{1} G_{3} \, + 4G_{4}^{2} } \right)} \right)\beta } \right) \hfill \\ + \,e^{{\frac{{H\left( {\kappa \, + \,G_{1} \, + \,G_{2} } \right)^{2} \beta }}{2\kappa }}} \sqrt {2\pi } \left( \begin{gathered} \kappa^{2} \left( {8\, + \,3q} \right)\, - \,2H\kappa \left( {G_{1}^{2} \, + \,G_{2}^{2} \, + \,2G_{1} \left( {G_{2} \, - \,\kappa G_{3} } \right)\, - \,4\kappa G_{4}^{2} } \right)q\beta \hfill \\ + \,H^{2} \left( {G_{1}^{2} \, + \,G_{2}^{2} \, + \,2G_{1} \left( {G_{2} \, - \,\kappa G_{3} } \right)\, - \,4\kappa G_{4}^{2} } \right)^{2} q\beta^{2} \, \hfill \\ - \,e^{{\frac{{H\left( {\kappa \, + \,G_{1} \, + \,G_{2} } \right)^{2} \beta }}{2\kappa }}} \sqrt {2\pi } \left( \begin{gathered} \kappa^{2} \left( {8\, + \,3q} \right) - \,2H\kappa \left( {G_{1}^{2} \, + \,G_{2}^{2} \, + \,2G_{1} \left( {G_{2} \, - \,\kappa G_{3} } \right)\, - \,4\kappa G_{4}^{2} } \right)q\beta \hfill \\ + \,H^{2} \left( {G_{1}^{2} \, + \,G_{2}^{2} \, + \,2G_{1} \left( {G_{2} \, - \,\kappa G_{3} } \right)\, - \,4\kappa G_{4}^{2} } \right)^{2} q\beta^{2} \hfill \\ Erf\left[ {\frac{{H\left( {\kappa \, + \,G_{1} \, + \,G_{2} } \right)\beta }}{{\sqrt {2H\kappa \beta } }}} \right] \hfill \\ \end{gathered} \right)\, \hfill \\ \end{gathered} \right) \hfill \\ \end{gathered} \right) \hfill \\ \end{gathered}$$

where the parameters $G_{1} ,\,\,G_{2} ,\,\,G_{3} ,\,\,G_{4} \,\,{\text{and}}\,\,H$ are defined in Eq. (11) above.

With the help of Eqs. (17) and (18) , other q-deformed thermodynamic properties in superstatistics regime can be obtained using the expressions below:

19 $$\left. \begin{gathered} F_{q} \left( \beta \right)\, = \, - \,\frac{1}{\beta }\,\ln \,Z_{q} \left( \beta \right)\,;\,\,\,S_{q} \left( \beta \right)\, = \, - \,k_{B} \frac{{\partial F_{q} \left( \beta \right)}}{\partial \beta }\,; \hfill \\ U_{q} \left( \beta \right)\, = \, - \,\frac{{\partial \ln \,Z_{q} \left( \beta \right)}}{\partial \beta }\,\,;\,\,C_{q} \left( \beta \right)\, = \,k_{B} \,\frac{{\partial \ln \,Z_{q} \left( \beta \right)}}{\partial \beta }\,. \hfill \\ \end{gathered} \right].$$

## Results and discussion

**Figure 1:**
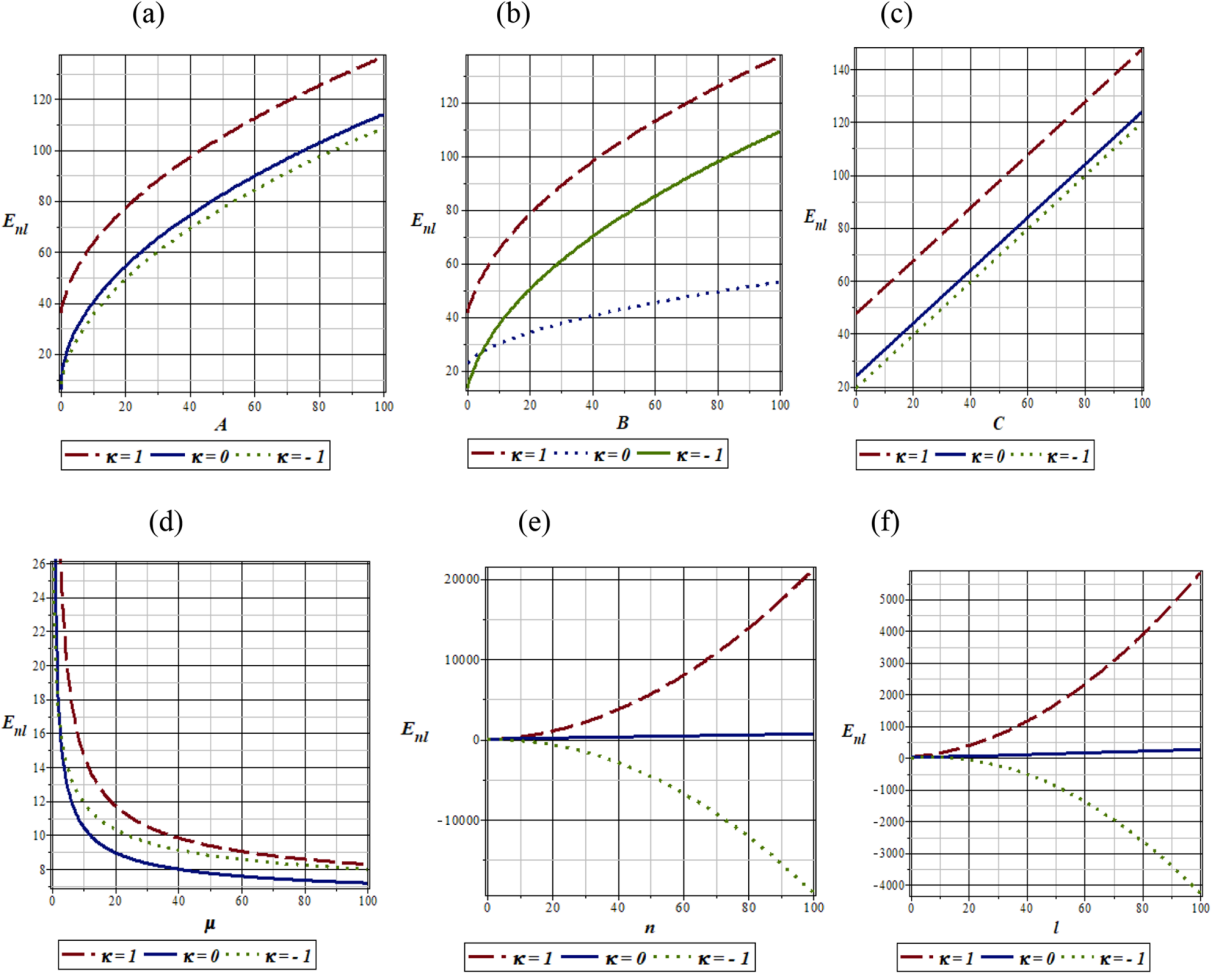
(**a**) Variation of energy eigenvalues of quantum pseudoharmonic oscillator with potential parameter ‘A’ for selected $\kappa$-values, (**b**) Variation of energy eigenvalues of quantum pseudoharmonic oscillator with potential parameter ‘B’ for selected $\kappa$-values, (**c**) Variation of energy eigenvalues of quantum pseudoharmonic oscillator with potential parameter ‘C’ for selected $\kappa$-values, (**d**) Variation of energy eigenvalues of quantum pseudoharmonic oscillator with reduced mass $^{\prime}\mu ^{\prime}$ for selected $\kappa$-values, (**e**) Variation of energy eigenvalues of quantum pseudoharmonic oscillator with radial quantum number ‘*n*’ for selected $\kappa$-values, (**f**)Variation of energy eigenvalues of quantum pseudoharmonic oscillator with orbital quantum number *‘l’* for selected $\kappa$-values.

In Fig. 1, we consider the variation of energy eigenvalues of the quantum pseudoharmonic oscillator given in Eqs. (6) and (7), with different potential parameters and quantum numbers. It is observed that the energy eigenvalues increases with an increase in potential parameters values for the selected $\kappa$ values (as seen in Fig. 1a,b and c respectively). In Fig. 1d, the energy eigenvalues decrease monotonously with increase in the reduced mass for the various $\kappa$ values. It is also observed that there exists an insignificant increase in energy eigenvalues as both principal quantum number and angular momentum quantum number increases, in the absence of the $\kappa$ values (see Fig. 1e and f, respectively). In the presence of the kappa values, the energy eigenvalues increases with an increase in both principal quantum number and angular momentum quantum number, for $\kappa \,\, = \,\,1$. For $\kappa \,\, = \,\, - \,1$, the energy eigenvalues decrease with increase in both principal quantum number, $n$ and angular momentum quantum number, $l$. Figure 1a–d physically describe the vibrational mode of pseudoharmonic oscillator atoms. Here both the vibrational and translational energies of the atom increases monotonically with an increase in the potential parameter.

**Figure 2:**
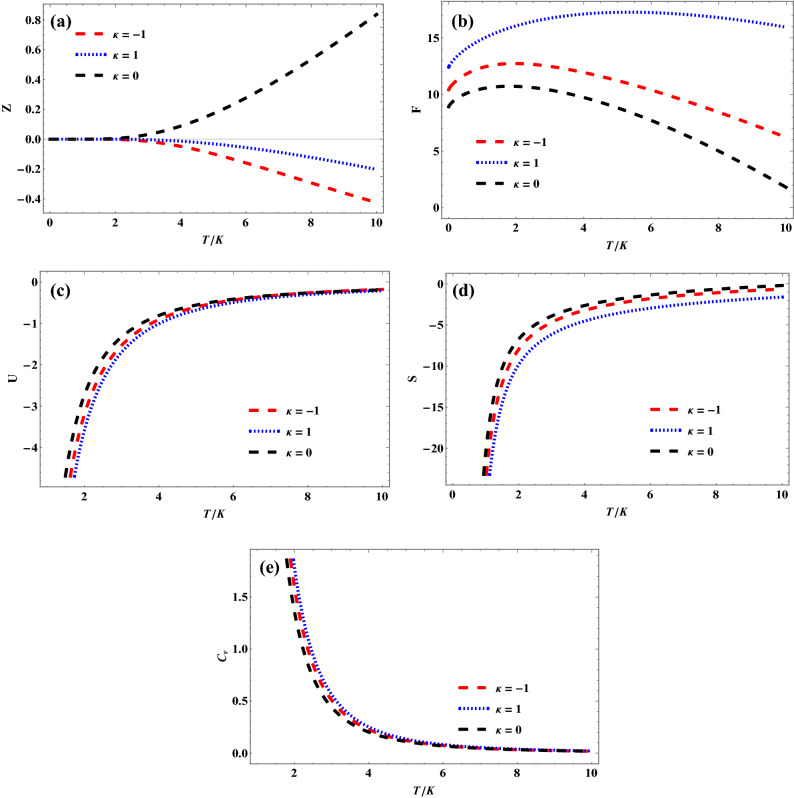
(**a**) Variation of partition function of quantum pseudoharmonic oscillator with temperature for selected $\kappa$-values. (**b**) Variation of free energy of quantum pseudoharmonic oscillator with temperature for selected $\kappa$-values. (**c**) Variation of internal energy of quantum pseudoharmonic oscillator with temperature for selected $\kappa$-values. (**d**) Variation of entropy of quantum pseudoharmonic oscillator with temperature for selected $\kappa$-values. (**e**) Variation of specific heat capacity of quantum pseudoharmonic oscillator with temperature for selected $\kappa$-values.

Figure 2 shows the variation of thermodynamic properties of quantum pseudoharmonic oscillator with temperature, for various $\kappa$ values. The thermodynamic properties expressions employed are given in Eqs. (9), (10) and (12). In Fig. 2a, the partition function of quantum pseudoharmonic oscillator remains constant for certain temperature values. As the temperature is enhanced further, the partition function for $\kappa \,\, = \,\,0$ increases, but the partition functions for both $\kappa \,\, = \,\,1,\,\, - \,1$ decreases. In Fig. 2b, the free energy increases first and later decreases monotonously as temperature increases, for the selected $\kappa$ values. Figure 2c shows a monotonous increase in internal energy as the temperature is enhanced, for the various $\kappa$ values selected. The internal energy plots tend to converge as the temperature values increases. The same trend is observed in Fig. 2d as entropy varies with temperature. Here, the entropy plots are seen to diverge as the temperature is enhanced more. In Fig. 2e, the specific heat capacity of quantum pseudoharmonic oscillator is seen to decrease with increase in temperature for the selected $\kappa$ values.

**Figure 3:**
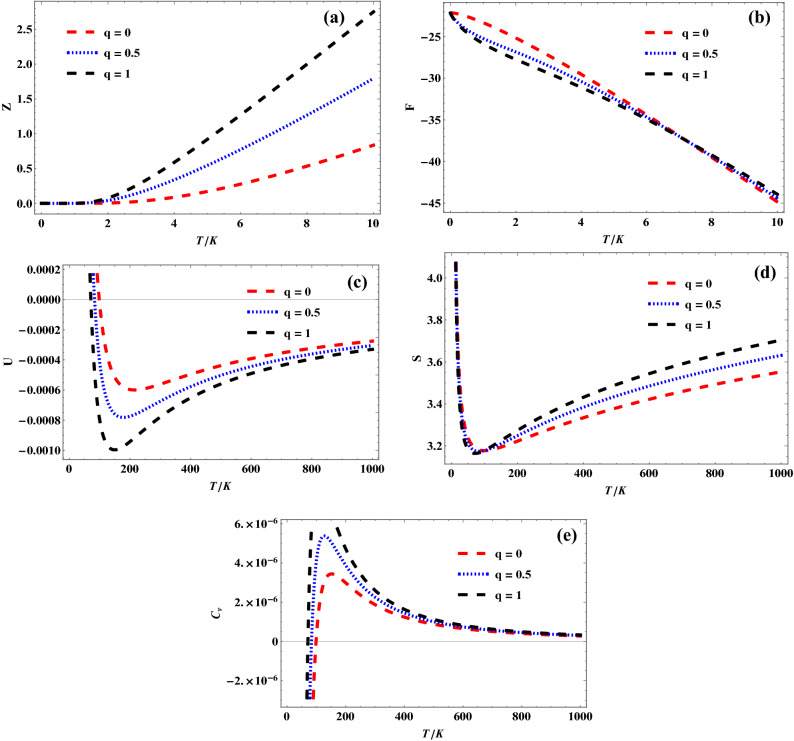
(**a**) Variation of partition function of quantum pseudoharmonic oscillator with temperature for selected *q*-values with $\kappa$ = 0. (**b**) Variation of free energy of quantum pseudoharmonic oscillator with temperature for selected *q*-values with $\kappa$ = 0. (**c**) Variation of internal energy of quantum pseudoharmonic oscillator with temperature for selected *q*-values with $\kappa$ = 0. (**d**) Variation of entropy of quantum pseudoharmonic oscillator with temperature for selected *q*-values with $\kappa$ = 0. (**e**) Variation of specific heat capacity of quantum pseudoharmonic oscillator with temperature for selected *q*-values with $\kappa$ = 0.

**Figure 4:**
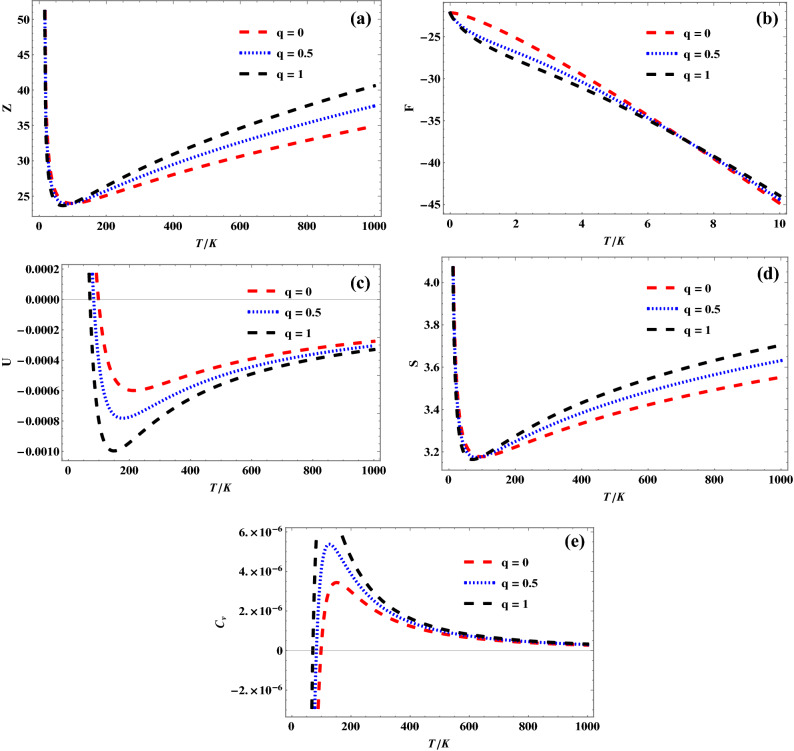
(**a**) Variation of partition function of quantum pseudoharmonic oscillator with temperature for selected *q*-values with $\kappa$ = 1. (**b**) Variation of free energy of quantum pseudoharmonic oscillator with temperature for selected *q*-values with $\kappa$ = 1. (**c**) Variation of internal energy of quantum pseudoharmonic oscillator with temperature for selected *q*-values with $\kappa$ = 1. (**d**) Variation of entropy of quantum pseudoharmonic oscillator with temperature for selected *q*-values with $\kappa$ = 1. (**e**) Variation of specific heat capacity of quantum pseudoharmonic oscillator with temperature for selected *q*-values with $\kappa$ = 1.

**Figure 5:**
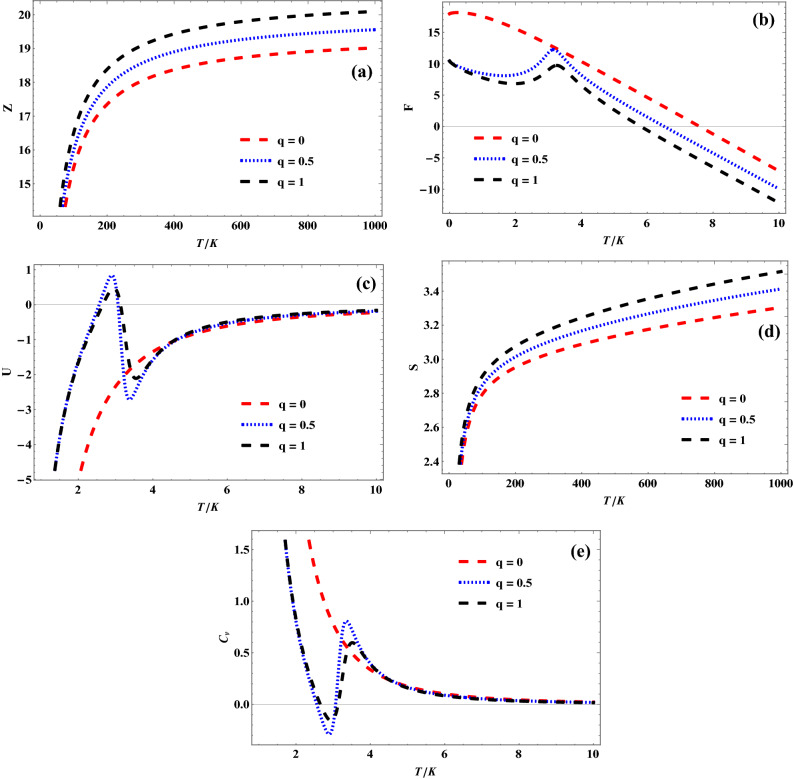
(**a**) Variation of partition function of quantum pseudoharmonic oscillator with temperature for selected *q*-values with $\kappa$ =− 1. (**b**) Variation of free energy of quantum pseudoharmonic oscillator with temperature for selected *q*-values with $\kappa$ = − 1. (**c**) Variation of internal energy of quantum pseudoharmonic oscillator with temperature for selected *q*-values with $\kappa$ = − 1. (**d**) Variation of entropy of quantum pseudoharmonic oscillator with temperature for selected *q*-values with $\kappa$ = − 1. (**e**) Variation of specific heat capacity of quantum pseudoharmonic oscillator with temperature for selected *q*-values with $\kappa$ = − 1.

Figures 3, 4 and 5 show the variation of different *q*-deformed superstatistics properties of quantum pseudoharmonic oscillator with temperature for selected $\kappa$ values, respectively. In Fig. 3a, the *q*-deformed partition function first remains constant and later increases sharply with increase in temperature, for the selected *q*-values. In Fig. 3b, the *q*-deformed free energy decreases sharply with increase in temperature. It is seen that the *q*-deformed free energy plots for the selected *q*-values tend to converge as the temperature is enhanced more. Figure 3c shows a sharp decrease in *q*-deformed internal energy for a specific temperature, corresponding to a unique *q*-value. As the temperature is enhanced, the *q*-deformed internal energy increases monotonously and tends to converge at a higher temperature value. This same trend is observed as *q*-deformed entropy varies with temperature in Fig. 3d. But the *q*-deformed entropy plots are seen to diverge at higher temperature values. In Fig. 3e, it is seen that the *q*-deformed specific heat capacity increases at a unique temperature value, corresponding to the selected *q*-values. As the temperature is enhanced, the *q*-deformed specific heat capacity plots are seen to decrease monotonously. The *q*-deformed specific heat capacity plots are seen to converge at much enhanced temperature values. The trend obtained in Figs. 4a–e for $\kappa \,\, = \,\,1$ are similar to that obtained in Figs. 3a–e for $\kappa \,\, = \,\,0$, as shown below:

Figure 5 show the variation of different *q*-deformed superstatistics properties of quantum pseudoharmonic oscillator with temperature for $\kappa \,\, = \,\, - \,1$. In Fig. 5a, the *q*-deformed partition function increases monotonously with increase in temperature, for the selected *q*-values. In Fig. 5b, the *q*-deformed free energy decreases sharply with increase in temperature for $q\,\, = \,\,0$. It is also observed that there exists an increase in the *q*-deformed free energy plots for $q\,\, = \,\,0.5,\,\,1$ and later a sharp decrease as the temperature increases. Figure 5c shows a monotonous increase in *q*-deformed internal energy as temperature increases for $q\,\, = \,\,0$. In the presence of the *q*-values, the *q*-deformed internal energy plots first increases, later decreases sharply and finally increases as the temperature is enhanced. It is seen that the entire *q*-deformed specific heat capacity plots converges at enhanced temperature values. Figure 5d shows a monotonous increase in the *q*-deformed entropy plots for the selected *q*-values, as temperature is increased. In Fig. 5e, it is seen that the *q*-deformed specific heat capacity for $q\,\, = \,\,0$ decreases monotonously as temperature is increased. In the presence of the *q*-values, the *q*-deformed specific heat capacity plots first decreases, later increases sharply and finally decreases as the temperature is enhanced. It is seen that the entire q-deformed specific heat capacity plots converge at enhanced temperature values.

## Concluding remarks

In this study, we have obtained the energy spectra of quantum pseudoharmonic oscillator in the curved space using the Nikiforov Uvarov method and used the energy spectra to obtain the partition function and other thermodynamic properties as a function of temperature both in the presence and absence of curvature parameter. With the help of the generalized Boltzmann factor of the modified Dirac delta distribution, the *q*-deformed superstatistics properties of quantum pseudoharmonic oscillator were obtained in the presence and absence of $\kappa$ parameters. In Fig. 2a, it can be observed that the trend of the partition function is largely affected by the curvature parameter ( $\kappa$). However, When $\kappa = 0$, the partition function returns to the conventional curve as observed in many existing literatures as shown in Fig. 3a. The monotonic increment in the variation of the energy against the potential parameter clearly described the vibrational and rotational energies of pseudoharmonic oscillator atoms. The variations of energy eigenvalues of the quantum pseudoharmonic oscillator with different potential parameters and quantum numbers were discussed extensively for the three values of $\kappa$ parameters. In addition, the variations of the thermodynamic and superstatistics properties with temperature have also been discussed in the presence and absence of both the $\kappa$ parameters and deformation parameters, respectively. It can be deduced that the ordinary statistics is obtained when the deformation parameter becomes zero in the superstatistics regime.

## Data Availability

Data will be available on request from the submission author: Dr. Ituen B. Okon through the email: ituenokon@uniuyo.edu.ng.
